# The psychological impact of Covid on health care professionals during the third wave

**DOI:** 10.1192/j.eurpsy.2023.889

**Published:** 2023-07-19

**Authors:** M. Theodoratou, A. Potoglou, A. Tamiolaki, A. Kalaitzaki

**Affiliations:** 1 Social Sciences, Hellenic Open University, Patras; 2 Health Sciences, Hellenic Mediterannean University, Herakleion, Greece

## Abstract

**Introduction:**

The COVID-19 pandemic is a healthcare crisis, with unprecedented impact on healthcare services, notable morbidity and mortality of the public and healthcare workers, economic impact and significant psychosocial impact. Besides, this pandemic has had a profound negative effect on the mental health of people worldwide, particularly among those who are faced with combating the virus.

**Objectives:**

The aim of this research was to examine the impact of the COVID-19 pandemic on healthcare workers’ mental health (HCWs), as they are on the front line of the pandemic.

**Methods:**

An internet-based questionnaire was created including the following scales: (1)Posttraumatic Stress Disorder Checklist (PCL-5), (2) Secondary Traumatic Stress Scale (STSS) (3) Quality of Professional Life (ProQOL) (4) Post-Traumatic Growth Inventory, (PTGI), (5) Brief Resilience Scale (BRS), (6) Brief Orientation to Problems Experienced Inventory (Brief Cope)], (7) Self-Compassion Scale (SCS-SF). It was distributed to 120 Greek health professionals, including nurses, doctors, midwives and physiotherapists.

**Results:**

Most of the participants were female with an average age of 46 years. HCWs had low levels of both primary and secondary traumatic stress . They presented post-traumatic growth in the dimension of relationship with others. They used predominantly the coping strategies of positive reframing, acceptance of the situation, venting, and instrumental support.

Females had statistically significant higher levels of post-traumatic growth, better quality of life, and used more positive coping strategies compared to males. Humor and acceptance were coping strategies used mainly by physicians. Nurses and midwives had worse quality of professional life potentially due to increased workload. HCWs with more functional ways of coping were more resilient and seemed to have better quality of life, such as higher compassion, satisfaction, lower burnout, and lower post-traumatic stress.

**Conclusions:**

The experience of the COVID-19 pandemic highlights the need to implement some strategies to protect health care workers’ mental health and to take extensive prevention measures in highly stressful situations.Further research is needed to clarify the long-term negative and positive psychological effects of the pandemic on healthcare personnel’s mental health.

**Disclosure of Interest:**

None Declared

